# Critical illness polyneuropathy in ICU patients is related to reduced motor nerve excitability caused by reduced sodium permeability

**DOI:** 10.1186/s40635-016-0083-4

**Published:** 2016-05-20

**Authors:** Susanne Koch, Jeffrey Bierbrauer, Kurt Haas, Simone Wolter, Julian Grosskreutz, Friedrich C. Luft, Claudia D. Spies, Jens Fielitz, Steffen Weber-Carstens

**Affiliations:** Department of Anesthesiology and Intensive Care Medicine, Campus Virchow-Klinikum and Campus Charité Mitte, Charité-Universitätsmedizin Berlin, Augustenburger Platz 1, 13353 Berlin, Germany; Klinik für diagnostische und interventionelle Radiologie und Nuklearmedizin, Klinikum Esslingen GmbH, Hirschlandstraße 97, 73730 Esslingen a.N, Germany; Neurology, Universitätsklinikum Jena, Jena, Germany; Experimental and Clinical Research Center, Charité - Universitätsmedizin Berlin, Max Delbrück Center for Molecular Medicine in the Helmholtz Association, Jena, Germany; Heart Center Brandenburg and Medical School Brandenburg (MHB), Bernau, Germany; Berlin Institute of Health (BIH), Berlin, Germany

**Keywords:** Critical illness polyneuropathy, Critical illness myopathy, Motor nerve excitability, Intensive care unit acquired weakness, Sepsis

## Abstract

**Background:**

Reduced motor and sensory nerve amplitudes in critical illness polyneuropathy (CIP) are characteristic features described in electrophysiological studies and due to dysfunction of voltage-gated sodium channels. Yet, faulty membrane depolarization as reported in various tissues of critically ill patients may cause reduced membrane excitability as well. The aim of this study was to compare the pathophysiological differences in motor nerve membrane polarization and voltage-gated sodium channel function between CIP patients and critically ill patients not developing CIP during their ICU stay (ICU controls).

**Methods:**

ICU patients underwent electrophysiological nerve conduction studies and were categorized as either ICU controls or CIP patients. Subsequently, excitability parameters were recorded as current-threshold relationship, stimulus-response behavior, threshold electrotonus, and recovery of excitability from the abductor pollicis brevis following median nerve stimulation.

**Results:**

Twenty-six critically ill patients were enrolled and categorized as 12 ICU controls and 14 CIP patients. When compared to 31 healthy subjects, the ICU controls exhibited signs of membrane depolarization as shown by reduced superexcitability (*p* = 0.003), depolarized threshold electrotonus (*p* = 0.007), increased current-threshold relationship (*p* = 0.03), and slightly prolonged strength-duration time constant. In contrast, the CIP patients displayed a significantly reduced strength-duration time constant (*p* < 0.0001), which indicates an increased inactivation of voltage-gated sodium channels.

**Conclusions:**

Abnormal motor nerve membrane depolarization is a general finding in critically ill patients whereas voltage-gated sodium channel dysfunction is a characteristic of CIP patients.

**Electronic supplementary material:**

The online version of this article (doi:10.1186/s40635-016-0083-4) contains supplementary material, which is available to authorized users.

## Background

Critically ill patients with systemic inflammatory response syndrome and multiple organ failure frequently develop muscle weakness due to critical illness myopathy and/or critical illness polyneuropathy (CIP) [1]. This weakness is caused by failure of muscle fibers and motor nerves to generate action potentials [2]. One of the primary affections is loss of membrane excitability [3, 4]. Loss of membrane excitability can either be caused by pathological membrane depolarization or by inactivation of voltage-gated sodium channels. So far, there are no in vivo studies in critically ill patients investigating these mechanisms. In an animal CIP model, inactivation of motor-nerve voltage-gated sodium channels was key in loss of membrane excitability [5]. On the other hand, faulty membrane depolarization was reported in various tissues of critically ill patients including muscle fibers, monocytes, and platelet mitochondria [6–8]. Importantly, motor-neuron excitability testing showed that CIP patients featured membrane depolarization after their discharge from the ICU [9]. However, membrane polarization in motor nerves of critically ill patients has received less attention. Moreover, the relationship between membrane depolarization and motor-nerve excitability in critically ill patients is poorly defined in general and particularly in CIP patients. This study tested the hypothesis that membrane depolarization is a general feature of critically ill patients, whereas inactivation of voltage-gated sodium channels is related to loss of membrane excitability in CIP patients, developing muscle weakness.

## Methods

The institutional review board of the Charité approved this study (ISRCTN77569430), and written informed consent of legal proxies was obtained. We screened ICU patients requiring mechanical ventilation on three of five consecutive days within the first week. Conventional nerve conduction studies were performed within 14 days by portable 2-Channel Keypoint Medtronic equipment (Skovlunde, Denmark) [1]. We performed sensory and motor nerve conduction studies using surface electrodes as follows: sensory nerve conduction velocity and sensory nerve action potentials of the sural nerve/median nerve followed by motor nerve conduction velocity and compound muscle action potential after nerve stimulation of the median/peroneal/tibial nerve (neCMAP). Nerve conduction studies were categorized according to the normal values of the neurophysiological laboratory of the Charité. Electromyography was performed to assess spontaneous activity using concentric needle electrodes in the extensor digitorum communis muscle and tibialis anterior muscles. To assess compound muscle action potential after direct muscle stimulation (dmCMAP), we placed a conventional stimulating surface electrode longitudinally over the muscle fibers just proximal to the distal tendon insertion. For recordings of dmCMAP, we used disposable concentric needle electrodes (length 25 or 37 mm; diameter 0.46 mm) and stimulated the muscle with gradually increasing strength (from 10 to 100 mA) using pulses of 0.1 ms in a duration delivered at 1 Hz. The recording electrode was placed 15–50 mm proximal to the stimulating electrode, guided by a muscle twitch. If there was no twitch visible, the recording electrode was placed in four different directions in order not to miss small amplitudes. In cases were no responses were obtained, the muscle was assumed to be inexcitable. The responses evoked were measured peak to peak. Muscle fiber action potentials were recorded using filter settings between 500 Hz and 10 kHz. Whenever possible, we examined the tibialis anterior muscle in the lower limb and the extensor digitorum communis muscle in the upper limb. Critical illness myopathy was diagnosed according to standard criteria, including a dmCMAP of less than <3 mV [10]. CIP was diagnosed according to standard criteria, including reduced motor and sensory nerve amplitudes [10]. Pathological spontaneous activity and reduced neCMAP were classified as unspecific and unable to differentiate between myopathy or neuropathy since it is a typical finding in both features. Patients without any pathologic features in electrophysiological testing consistent with myopathy or polyneuropathy were classified as ICU control.

Patients featuring isolated CIM were not further evaluated. All CIP patients presented critical illness myopathy criteria in electrophysiological assessment as well. Limb temperature was kept at >32 °C during electrophysiological exams and during the excitability test.

An automated protocol (Threshold tracking; Qtrac version 28/10/2009; Institute of Neurology, Queen Square, London, TRONDF) was used to measure excitability parameters (current-threshold relationship, stimulus-response relationship, threshold electrotonus, and recovery of excitability) within 7 to 10 days after ICU admission [11]. This multiple excitability protocol assesses motor-nerve threshold following different conditioning stimuli [12]. The median nerve was stimulated with surface electrodes at the wrist, and motor action potentials were recorded from the abductor pollicis brevis muscle.

1. The current-threshold relationship (I/V) was measured with 1-ms pulses following sub-threshold polarizing currents of a 200-ms duration, which were altered in steps of 10 % between +50 % (depolarizing) and −100 % (hyperpolarizing) of the control threshold.
2. Stimulus-response relationship was generated using current impulses of 0.2 and 1 ms. The peak 1-ms response was used to calculate the target response (set at 40 % of the supramaximal CMAP response). The ratio between stimulus-response curves of both stimuli was used to calculate rheobase and strength-duration time constant (SD_tc_).
3. Threshold electrotonus (TE) was measured by altering nerve excitability using prolonged sub-threshold polarizing currents of 100 ms duration set at 40 % of the control threshold currents and is defined as threshold changes occurring in response to sub-threshold depolarizing and hyperpolarizing pulses [7, 8]. Finally, TE will pass a phase of sub-excitability following the depolarizing conditioning current (=TEd^40^ (undershoot)) and a phase of superexcitability following the hyperpolarizing conditioning current (=TEh40 (overshoot)).
4. The recovery of excitability following a supramaximal conditioning stimulus was tested at 18 conditioning test intervals, decreasing from 200 to 2 ms in geometric progression.

Excitability parameters of critically ill patients were matched with data assessed in 31 healthy subjects (age 36 ± 9.5 years).

Data analysis has been carried out by QtracP software (Qtrac version 28/10/2009; Institute of Neurology, Queen Square, London), additionally using the modeling software “MEMFIT” included in the QtracP software by Bostock and colleagues to simulate the threshold tracking data [13–15]. Lab results were recorded during threshold tracking assessments in the ICU. Potassium administration over the first 2 weeks was averaged as means per day.

Physical examination of muscle strength was conducted using the Medical Research Council (MRC) score (range: 0 = no muscle contraction to 5 = normal strength) at ICU discharge [16]. Whenever possible, we examined three muscles in each limb, including the triceps, biceps brachii, and extensor digitorum muscles in the upper limbs and rectus femoris, the tibialis anterior as well as the gastrocnemius muscles in the lower limbs, respectively. In case of hindered circumstances (e.g., unilateral or central paresis, bone fractures, or fixateur externe), the affected muscles where not included in our MRC results. MRC scores are presented as average muscle strength of the muscles examined.

Statistical analysis tests were computed by SPSS, Version 19, Copyright© SPSS, Inc., Chicago, IL 60606, USA. We conducted non-parametric tests using the Mann-Whitney test for two independent samples, Kruskal-Wallis test for three or more independent samples, and Fisher’s test (chi-square test) for qualitative data. In case of small samples, greater differences in sample sizes, large but unbalanced groups, data sets containing ties, or sparse data, tests were carried out in an exact version. For analysis of correlations between lab parameters and excitability, we used the Spearman correlation coefficients.

## Results

**Fig. 1:**
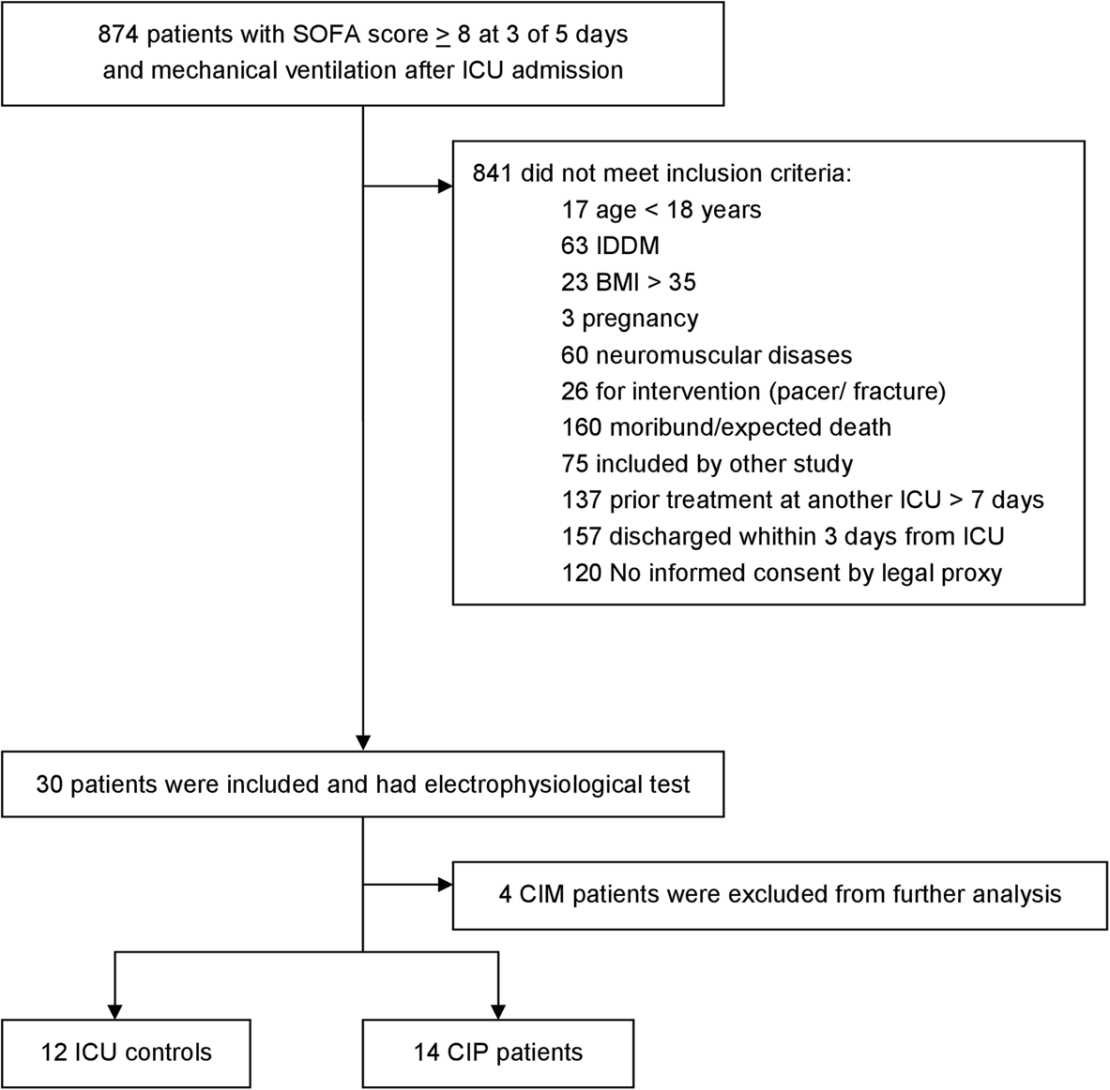
Study flow chart

Eight hundred seventy-four mechanically ventilated patients with a Sequential Organ Failure Assessment (SOFA) score ≥8 for three consecutive days in the first 5 days on ICU were screened in this prospective observational study. Eight hundred forty-one patients did not meet the inclusion criteria, and four patients were excluded from further analysis since they featured isolated CIM (Fig. 1). We enrolled and classified 26 ICU patients as either ICU controls (*n* = 12) or CIP patients (*n* = 14). Patient’s characteristics are shown in Table 1. Muscle strength was significantly reduced in the CIP patients compared to the ICU controls (*p* = 0.01) (Table1).

**Table 1:** Clinical characteristics

	ICU controls (*n* = 12)	CIP patients (*n* = 14)	*p* value
Age (years)	48.4 (11.4)	53.2 (14.8)	0.193
Gender (m/f)	8 / 4	11 / 3	0.66
BMI (kg/m^2^) on admission	29.6 (4.4)	27.0 (4.7)	0.25
Diagnosis on admission:			
ALI/ARDS	2	10	0.011
Sepsis	–	2	
Intracranial bleeding	7	1	
Multiple trauma	2	1	
Severe cardiac dysfunction/after resuscitation	1	–	
SAPS-II on admission	39.9 (14.5)	56.4 (17.8)	0.027
SOFA max. within ICU stay	11.4 (4)	13.2 (3.9)	0.179
MRC score (mean of 12 muscles assessed) at ICU discharge	4.7 (0.4)	3.1 (0.2)	0.01
ICU survival (yes/no)	11/1	8/5	0.16
ICU length of stay (days)	27.3 (10.8)	42.2 (30.5)	0.347

*p* value compares ICU control patients versus CIP patients, (Mann-Whitney *U*/Fischer’s exact test). Values are shown as mean (SD) or as absolute numbers/%

*BMI* body mass index, *ARDS* acute respiratory distress syndrome, *ALI* acute lung injury, *SAPS-II* simplified acute physiology score, *SOFA* Sequential Organ Failure Assessment, *MRC* medical research council, *ICU length of stay* intensive care unit length of stay.

**Fig. 2:**
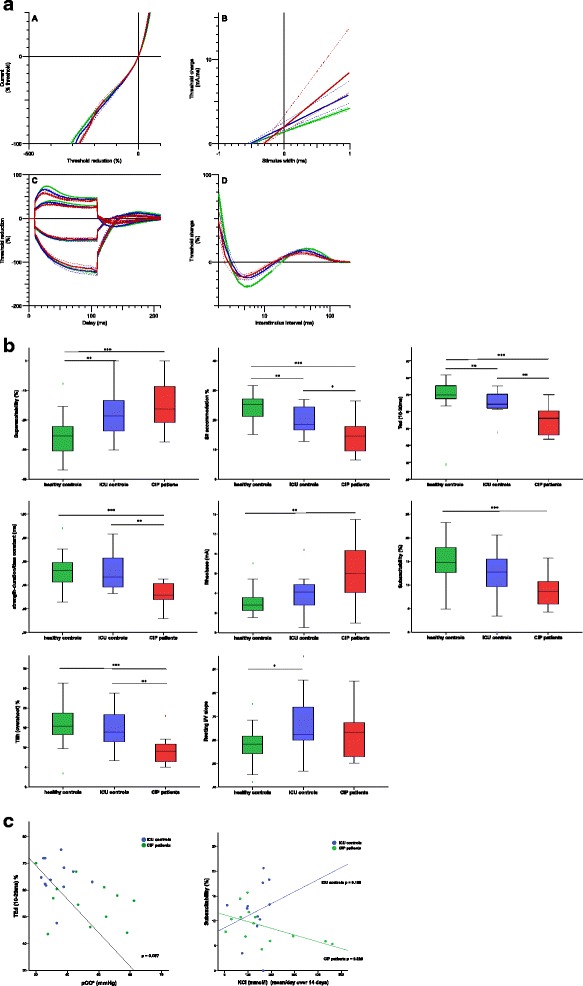
**a** Mean excitability data ± SE for 31 healthy subjects (*n* = 31, *green curve*), ICU controls (*n* = 12, *blue curve*), and CIP patients (*n* = 14, *red curve*). (*1*) I/V slope. (*2*) Stimulus-response relationship. (*3*) Threshold electrotonus. (*4*) Excitability recovery. **b** Correlation between healthy controls (*n* = 31, *green boxes*), ICU controls (*n* = 12, *blue boxes*), and CIP patients (*n* = 14, *red boxes*) for superexcitability %, TEd_(10–20 ms)_, S2 accommodation, resting I/V slope, strength-duration time constant, rheobase, sub-excitability, and TEh (overshoot). Differences between groups are indicated as *p < 0.05; **p < 0.01; ***p < 0.0001. **c** Correlation between laboratory parameters and excitability parameters in ICU controls (*n* = 12) and CIP patients (*n* = 14). pCO_2_ and TEd_(10–20 ms)_ (*p* = 0.007). KCl mmol/l infusion rate mean/day for the first 14 days on ICU and sub-excitability. Significant correlation in CIP patients (*p* = 0.026), contrary to ICU control patients (*p* = 0.159)

Significant differences of excitability parameters (Table 2) between the ICU controls and healthy subjects [reduced: superexcitability %, (*p* = 0.003); TEd^40^_(10–20ms)_, (*p* = 0.007); S2^40^ accommodation, (*p* = 0.006)] indicated membrane depolarization in the ICU controls. When compared to the healthy subjects, the CIP patients showed a different pathology [reduced: superexcitability %, (*p* < 0.0001); TEd^40^_(10–20ms)_; (*p* < 0.0001); S2^40^ accommodation, (*p* < 0.0001); TEh^40^_(10–20 ms)_, (*p* = 0.028); late sub-excitability, (*p* < 0.0001); SD_tc_, (*p* < 0.0001)], which coincides with an inactivation of voltage-gated sodium channels. Significant differences between the ICU controls and the CIP patients were observed for SD_tc_ (*p* = 0.03) and TEd40_(10–20ms)_ (*p* = 0.002); S2^40^ accommodation (*p* = 0.021) (Fig. 2a, b).

**Table 2:** Excitability parameters

	Healthy subjects (*n* = 31)	ICU controls (*n* = 12)	CIP patients (*n* = 14)	*p* ^a^ value	*p* ^b^ value	*p* ^c^ value
Temperature °C	33.94 (1.3)	34.5 (1.8)	34.8 (1.5)	0.399	0.075	0.705
(1) Current/threshold relationship						
Resting I/V slope	0.58 (0.01)	0.66 (0.04)	0.63 (0.03)	0.03	0.239	0.728
Minimum I/V slope	0.26 (0.01)	0.28 (0.02)	0.26 (0.02)	0.536	0.781	0.406
Hyperpol. I/V slope	0.4 (0.01)	0.31 (0.05)	0.44 (0.08)	0.225	0.263	0.079
(2) Stimulus response/strength duration						
Stimulus 50 % (mA)	4.66 (0.27)	5.52 (1.06)	8.6 (1.21)	0.282	0.001	0.075
SDtc (ms)	0.51 (0.02)	0.56 (0.08)	0.4 (0.07)	0.759	<0.0001	0.03
Rheobase (mA)	3.07 (0.22)	3.97 (0.58)	6.1 (0.87)	0.081	0.002	0.095
Stimulus-response slope	6.39 (0.29)	4.51 (0.51)	4.61 (0.38)	0.004	0.003	1
(3) Threshold electrotonus (%)						
TEd^40^_(10–20 ms)_	69.97 (1.69)	64.8 (2.04)	44.7 (11.1)	0.007	<0.0001	0.002
TEh^40^_(10–20 ms)_	−74.15 (1.76)	−76.92 (2.67)	−66.65 (3.74)	0.841	0.028	0.051
S2^40^ accommodation	24.99 (1.03)	20.1 (1.33)	14.77 (1.97)	0.006	<0.0001	0.021
TEd^40^_(90–100 ms)_	45.19 (1.5)	43.59 (1.64)	44.29 (2.58)	0.123	0.17	0.932
TEh^40^_(90–100 ms)_	−123.42 (3.37)	−121.91 (8.76)	−110.96 (7.61)	0.862	0.222	0.379
TEh^40^ overshoot	15.88 (0.84)	14.64 (1.42)	9.39 (1.12)	0.342	<0.0001	0.004
TEd^40^ undershoot	−18.75 (0.74)	−17.93 (1.38)	−11.58 (1.64)	0.752	0.001	0.009
(4) Recovery of excitability						
RPR (ms)	3.06 (0.08)	2.98 (0.45)	3.06 (0.18)	0.874	0.8	0.968
Superexcitability (%)	−25.69 (1.09)	−17.13 (2.74)	−14.39 (2.05)	0.003	<0.0001	0.403
Superexcitability at 7 ms	−23.92 (1.19)	−15.7 (2.41)	−11.05 (1.8)	0.001	<0.0001	0.12
Superexcitability at 5 ms	−26.4 (1.17)	−17.3 (2.85)	−14.46 (2.26)	0.004	<0.0001	0.501
Sub-excitability (%)	14.78 (0.91)	11.8 (1.7)	9.04 (0.92)	0.114	<0.0001	0.106

Values are given as mean ± SE

^a^Healthy subjects versus ICU control

^b^Healthy subjects versus CIP patients

^c^ICU control versus CIP patients

In the CIP patients, pCO_2_ (46.4 ± 9.8 versus 36.7 ± 4.8 mmHg; *p* = 0.009), HCO^3^ (29.4 ± 6.2 versus 23.8 ± 2.5 mmol/l; *p* = 0.031), and lactate (12.6 ± 4.8 versus 8.1 ± 2.6 mg/dl; *p* = 0.006) levels were significantly higher than in those of the ICU controls. Regarding all ICU patients, elevated pCO_2_ (*p* = 0.007) and lactate (*p* = 0.016) levels were significantly correlated with membrane depolarization [reduced TEd_(10–20 ms)_, S2^40^ accommodation] (Table 3/Fig. 2c). On average, the mean KCl administration was 131 mmol/l in ICU controls and 161 mmol/l in CIP patients (Additional file 1: Table S2). CIP showed a statistically significant negative correlation between reduced sub-excitability and elevated KCl administration. In contrast, ICU controls showed a direct correlation between increased sub-excitability and elevated KCl administration (Fig. 2c).

**Table 3:** Correlation between selected excitability parameters and lab results

	pCO^2^ mmHg	HCO^3^ mmol/l	Lactate mg/dl	K mmol/l	Na mmol/l
SDtc (ms)	0.075	0.41	0.102	0.993	0.543
Rheobase (mA)	0.203	0.875	0.066	0.097	0.728
TEd^40^_(10–20 ms)_	0.007**	0.2	0.016*	0.58	0.924
S2^40^ accommodation	0.175	0.215	0.013*	0.475	0.072
Superexcitability %	0.832	0.533	0.396	0.348	0.087
Late sub-excitability %	0.165	0.066	0.664	0.796	0.147

*SD*_*tc*_ strength duration time constant, *TE* threshold electrotonus, *RRP* relative refractory period, *HCO* ^*3*^ bicarbonate

* *p* values < 0.05; ** *p* values < 0.01

To help interpret these different changes in critically ill patients, we ran the modeling software MEMFIT included in the QtracP software [12]. Using MEMFIT, sodium currents of motor axons were modeled using the voltage clamp data of Schwarz and colleagues [13]. Further empirical parameter adjustments were made to improve the fit of recovery of excitability, SD_tc_, TE, and current-threshold-relationship in the healthy controls [14]. We ran MEMFIT to get the best fit of data recorded in the ICU controls and the CIP patients. Excitability measurements of ICU controls where best modeled by a two fold increase of fast K+ currents (Additional file 2: Figure S1/Table 4), whereas in the CIP patients, the differences in comparison to healthy controls were best modeled by a twofold reduction of Na+ permeability (Additional file 3: Figure S2/Table 4).

**Table 4:** MEMFIT results modeling excitability parameters for ICU controls and CIP patients

	ICU controls (%)	CIP patients (%)
P Na p (%) (percent of persistent Na)	51.87	80.4
P Na N (nodal sodium permeability)	46.37	72.49
G Kf l (internodal fast K conductance)	65.89	74.217
G Kf N (nodal fast K conductance)	57.71	73.52
G Ks N (nodal slow K conductance)	51.56	53.2
I pump NI (pump currents)	31.07	11.6

MEMFIT results modeling ICU controls and CIP patients excitability parameters showing discrepancy in percentage for sodium and potassium currents between healthy controls and ICU controls as well as healthy controls and CIP patients (please notice that in the modeling model, only the primary change is reliable)

## Discussion

By performing motor-nerve excitability tests in critically ill patients during their early ICU stay, we are the first group to demonstrate in vivo-reduced membrane excitability related to Na+ channel inactivation in CIP patients. Contrary, the ICU control patients without CIP showed motor axon membrane depolarization.

### Reduced membrane excitability in CIP patients during their ICU stay

Membrane depolarization as indicated by reduced superexcitability, TE, and elevated SD_tc_ in motor nerve excitability testing has been proposed as the primary, pathological sign in the CIP patients after ICU discharge [9]. In contrast, we observed significantly reduced SD_tc_ [beside reduced superexcitability, TE, and sub-excitability] indicating reduced membrane excitability. SD_tc_ depends on passive membrane properties, as well as on voltage-dependent Na+ conductance [17, 18]. Possible explanations for reduced SD_tc_ in motor axons could be structural changes, membrane hyperpolarization, or persistently decreased Na+ conductance [11, 17, 18]. Structural changes of the motor neurons are not likely, since nerve conduction velocity was normal and since it has been reported that biopsies of sensory nerves obtained from patients with CIP show no structural abnormalities [19]. Membrane hyperpolarization was not evident. We suggest that a decrease in persistent Na+ conductance accounts for the reduction of SD_tc_ in CIP patients. This hypothesis is confirmed by modeling our excitability data for CIP patients. The twofold decrease of Na+ current in CIP patients is in line with a nerve excitability study of patients with puffer fish—TTX [Na+ channel blocker] intoxication [17]—where a decrease of Na+ currents by a factor of two accounted for reduced superexcitability, sub-excitability, and TE and increased SD_tc_.

Our study confirms that dysfunction of voltage-gated Na+ channels are involved in the pathomechanism of reduced motor nerve membrane excitability in CIP patients. This hypothesis is supported by an animal model of CIP describing increased inactivation of voltage-gated sodium channels as an important contributor to reduced excitability [3]. Voltage-gated Na+ channel inactivation can be impaired by oxidative stress and endotoxins [20, 21], which coincides with significant elevated pCO_2_ levels in our CIP patients group compared to the ICU controls.

Our study also confirms that increased inactivation of voltage-gated Na+ channels shown in an animal model of CIP [3] causes reduced membrane excitability, which is a trigger for the development of muscle weakness [2].

Reduced TEd_(10–20 ms)_ and S2^40^ accommodation indicate membrane depolarization and were correlated with elevated blood lactate concentrations in all patients. As increased blood lactate concentration is a marker for illness severity in critically ill patients [22], our results are in line with studies showing that membrane depolarization in critically ill patients is directly correlated with severity of illness [6–8]. However, further studies are needed to elucidate the molecular mechanisms of our observations.

In contrast to our study, Z’Graggen and colleagues observed membrane depolarization of motor nerves in CIP patients [9]. However, these patients were examined 2–3 weeks after their ICU stay. According to the study of Haeseler and colleagues, dysfunction of sodium channels is related to endotoxin levels (LPS), yet patients in the post intensive care period should feature endotoxin levels close to zero [21]. Moreover, dysfunction of sodium channels has been reported to be associated with oxidative stress. Likewise, oxidative stress should be resolved after the ICU stay [20]. These important differences explain the different findings in the study of Z’Graggen and our study.

Early changes in membrane excitability during the ICU stay are very likely and have been shown in muscle as early as 6 h after the onset of porcine fecal peritonitis [23]. However, due to different excitability examination setups in muscle, it was impossible to differentiate between sodium channel dysfunction and/or membrane depolarization.

### K+ currents, sub-excitability, and membrane depolarization

According to the MEMFIT model, an increase in fast K+ currents causes the changes in the ICU controls. In the healthy subjects, elevated [K+]_o_ causes an increase of K+ current over the membrane, as demonstrated by a decrease in superexcitability [24, 25]. Late sub-excitability is the best indicator of [K+]_o_ from nerve excitability measurements, owing to the activation of slow K+ channels during the action potential [12]. But interestingly, even though K+ currents are elevated according to the model, there is reduced sub-excitability in critically ill patients. Furthermore, we did not find a correlation between [K+]_o_ and superexcitability or late sub-excitability. Interestingly, elevated KCl infusion rates were correlated to reduced late sub-excitability (*p* = 0.026) in the CIP patients, the opposite of what we observed in the ICU controls and what has been shown in the healthy subjects [25]. These paradox findings may be related to instable [K+]_o_ plasma levels in critically ill patients requiring potassium substitution in order to keep [K+]_o_ plasma concentration within the normal range. Further, it needs to be mentioned, that changes in sub-excitability/superexcitability are related to local potassium levels which may be different from local potassium levels in healthy subjects due to poor microcirculation in critically ill patients [26]. The inverse relation between the ICU controls and the CIP patients may be related to the significantly elevated pCO_2_ concentrations in CIP patients which will cause higher intracellular H+ levels and could finally induce reduced K+ current. It has been shown that prolonged ischemia causes membrane depolarization in sensory nerves, correlating with a “paradoxical” reduction of K+ currents by increasing intracellular acidosis [27]; however, those findings are still a matter of discussion and need further exploration.

### Limitations

Our study has limitations. All of our CIP patients featured concomitant critical illness myopathy, which may have influenced excitability results in motor axons. However, since conditioning currents applied in stimulus-response and current-threshold relationships and TE assessments would not be sensed by the innervated muscle fibers, these data should not have been affected by concomitant myopathy. Due to the explorative and non-confirmatory study design, we avoid to report a post hoc power analysis in the paper, as recommended by Hoenig and colleagues [28].

## Conclusions

We confirmed previous in vitro findings in vivo, showing that inactivation of motor axon voltage-gated Na+ channels is the primary contributor of muscle weakness in CIP patients. Additionally, we provided evidence that abnormal membrane depolarization in motor axons is a general finding in ICU patients corresponding to illness severity. Moreover, we observed a paradoxical membrane depolarization related to K+ equilibrium that could be related to intracellular acidosis; however, this still needs to be evaluated in future studies.

## Additional files

Additional file 1: Table S1. Electrophysiological data. neCMAP, nerve evoked compound muscle action potential amplitude; SNAP, sensory nerve action potential amplitude; dmCMAP, direct muscle evoked compound muscle action potential amplitude. *p* value compares ICU controls versus CIP patients (Mann-Whitney *U*). Values are given as mean ± SD. Table S2. Laboratory data *p* value compares ICU controls with CIP patients. pCO, partial arterial pressure of carbon dioxide; pO^2^, partial arterial pressure of oxygen; HCO_3_, bicarbonate; Na, sodium; K, potassium; Ca, calcium. (DOC 52 kb) Additional file 2: Figure S1. MEMFIT data showing best fit of current changes in % for ICU controls. GKfl, internodal fast K conductance; GKfN, nodal fast K conductance; GKsN, nodal slow K conductance, PNa p (%), percent of persistent Na; P Na N, nodal sodium permeability; GLkN, nodal leak conductance; IPumpNI, pump currents; GKsI internodal slow K conductance). (TIF 104 kb) Additional file 3: Figure S2. MEMFIT data showing best fit of current changes in % for critical illness polyneuropathy patients. GKfl, internodal fast K conductance; GKfN, nodal fast K conductance; GKsN, nodal slow K conductance; PNa p (%), percent of persistent Na; P Na N, nodal sodium permeability; GLkN, nodal leak conductance; IPumpNI, pump currents; GKsI, internodal slow K conductance). (TIF 112 kb)
