# Supplementary material for: Critical illness polyneuropathy in ICU patients is related to reduced motor nerve excitability caused by reduced sodium permeability
Source: Intensive Care Med Exp. 2016 May 20;4:10. doi: 10.1186/s40635-016-0083-4 (PMC4875580; doi:10.1186/s40635-016-0083-4)
Supplement: Additional file 1: Table S1. — Electrophysiological data. neCMAP, nerve evoked compound muscle action potential amplitude; SNAP, sensory nerve action potential amplitude; dmCMAP, direct muscle evoked compound muscle action potential amplitude. p value compares ICU controls versus CIP patients (Mann-Whitney U). Values are given as mean ± SD. Table S2. Laboratory data p value compares ICU controls with CIP patients. pCO, partial arterial pressure of carbon dioxide; pO2, partial arterial pressure of oxygen; HCO3, bicarbonate; Na, sodium; K, potassium; Ca, calcium. (DOC 52 kb) [file 40635_2016_83_MOESM1_ESM.doc]

**Supplement Figure legends**

**Figure s1** MEMFIT data showing best fit of current changes in % for ICU controls (GKfl = Internodal fast K conductance; GKfN = Nodal fast K conductance; GKsN = Nodal slow K conductance; PNa p (%) = percent of persistent Na; P Na N = Nodal sodium permeability; GLkN = Nodal Leak conductance; GBB = ???; IPumpNI = pump currents; GH = ???; GKsI = Internodal slow K conductance).

**Figure s2** MEMFIT data showing best fit of current changes in % for critical illness polyneuropathy patients (GKfl = Internodal fast K conductance; GKfN = Nodal fast K conductance; GKsN = Nodal slow K conductance; PNa p (%) = percent of persistent Na; P Na N = Nodal sodium permeability; GLkN = Nodal Leak conductance; GBB = ???; IPumpNI = pump currents; GH = ???; GKsI = Internodal slow K conductance).

**Table s1 Electrophysiological data**

|  | **Nerve / Muscle** | **ICU – controls**  **(n=12)** | **CIP**  **(n=14)** | **p-value** |
| --- | --- | --- | --- | --- |
| **neCMAP (mV)** | N. tibialis (Ext. hall. brev.) | 4.3 + 2.6 | 1.3 + 1.8 | 0.001 |
|  | N. peroneus (Ext. dig. brev.) | 3.2 + 2.1 | 1.3 + 1.4 | 0.018 |
| **SNAP (µV)** | N. suralis | 9.1 + 4.5 | 1.6 + 1.6 | <0.0001 |
| **dmCMAP (mV)** | Tibialis anterior muscle | 4.2 + 1 | 0.8 + 0.9 | <0.0001 |

**Table s1 Electrophysiological data.** neCMAP, nerve evoked compound muscle action potential amplitude, SNAP, sensory nerve action potential amplitude, dmCMAP, direct muscle evoked compound muscle action potential amplitude. p-value compares ICU controls versus CIP patients (Mann-Whitney U). Values are given as mean ± SD.

**Table s2 Laboratory data**

|  | **ICU – controls**  **(n=12)** | **CIP patients**  **(n=14)** | **p-value** |
| --- | --- | --- | --- |
| **pH (Norm: 7.34-7.44)** | 7.42 + 0.05 | 7.41 + 0.07 | 0.527 |
| **pCO² mmHg (Norm: 32-46 mmHg)** | 36.7 + 4.8 | 46.4 + 9.82 | 0.009 |
| **pO² mmHg (Norm; 71-104 mmHg)** | 106.6 + 28 | 87.8 + 30.1 | 0.145 |
| **HCO³ mmol/l (Norm 21-28 mmol/l)** | 23.8 + 2.5 | 29.4 + 6.2 | 0.031 |
| **Lactate mg/dl (Norm 6-18 mg/dl)** | 8.1 + 2.6 | 12.6 + 4.8 | 0.006 |
| **Glucose mg/dl (Norm 56-110mg/dl)** | 117.8 + 18.7 | 121.6 + 17.1 | 0.631 |
| **Na mmol/l (Norm 134-145 mmol/l)** | 139.9 + 4.6 | 145.6 + 8.9 | 0.118 |
| **K mmol/l (Norm 3.4-5.2 mmol/l)** | 4.3 + 0.3 | 4.2 + 0.4 | 0.705 |
| **Ca mmol/l (Norm 2.15-2.65 mmol/l)** | 1.15 + 0.03 | 1.93 + 2.81 | 0.347 |
| **KCl mean / day first 14 ICU days** | 132.56 + 53.36 | 161,08 + 140.43 | 0.667 |

**Table s2 Laboratory data** p-value compares ICU controls with CIP patients. pCO = partial arterial pressure of carbon dioxide, pO² partial arterial pressure of axygen, HCO_3_ = bicarbonate, Na = sodium, K = potassium, Ca = calcium.
