# Supplementary figures and images for: Critical illness polyneuropathy in ICU patients is related to reduced motor nerve excitability caused by reduced sodium permeability
Source: Intensive Care Med Exp. 2016 May 20;4:10. doi: 10.1186/s40635-016-0083-4 (PMC4875580; doi:10.1186/s40635-016-0083-4)

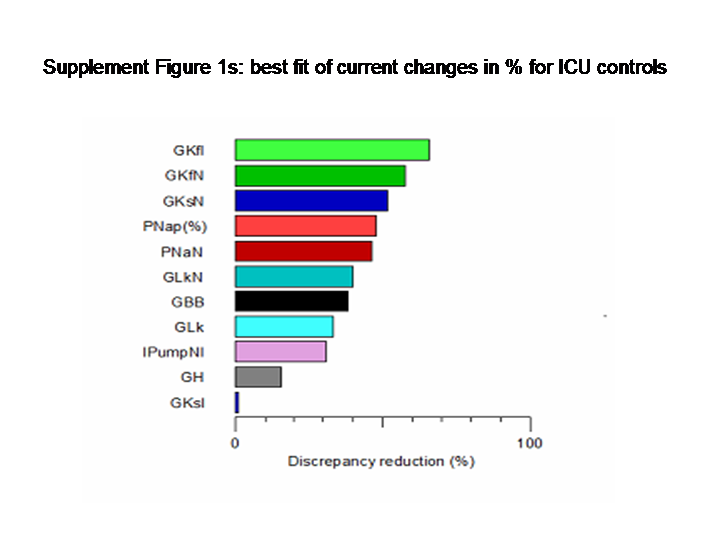

Supplement: Additional file 2: Figure S1. — MEMFIT data showing best fit of current changes in % for ICU controls. GKfl, internodal fast K conductance; GKfN, nodal fast K conductance; GKsN, nodal slow K conductance, PNa p (%), percent of persistent Na; P Na N, nodal sodium permeability; GLkN, nodal leak conductance; IPumpNI, pump currents; GKsI internodal slow K conductance). (TIF 104 kb) [file 40635_2016_83_MOESM2_ESM.tif]

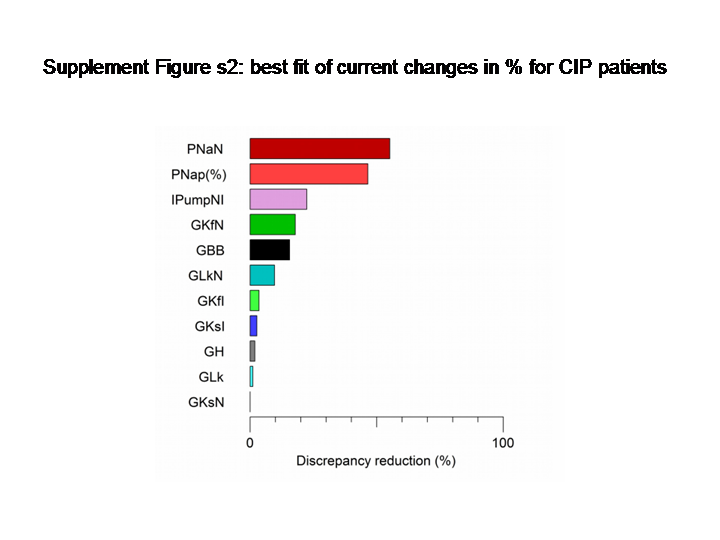

Supplement: Additional file 3: Figure S2. — MEMFIT data showing best fit of current changes in % for critical illness polyneuropathy patients. GKfl, internodal fast K conductance; GKfN, nodal fast K conductance; GKsN, nodal slow K conductance; PNa p (%), percent of persistent Na; P Na N, nodal sodium permeability; GLkN, nodal leak conductance; IPumpNI, pump currents; GKsI, internodal slow K conductance). (TIF 112 kb) [file 40635_2016_83_MOESM3_ESM.tif]
